# Seeing Through Each Other’s Hearts: Inferring Others’ Heart Rate as a Function of Own Heart Rate Perception and Perceived Social Intelligence

**DOI:** 10.1007/s42761-022-00151-4

**Published:** 2022-11-02

**Authors:** Irena Arslanova, Alejandro Galvez-Pol, James Kilner, Gianluca Finotti, Manos Tsakiris

**Affiliations:** 1 Department of Psychology, Royal Holloway, University of London, London, UK; 2 Psychology Department, University of Balearic Islands, Palma, Spain; 3 Institute of Neurology, University College London, London, UK; 4 Centre for the Politics of Feeling, School of Advanced Study, University of London, London, UK

**Keywords:** Heartbeat perception, Cardiac state, Interception, Mentalizing, Interoceptive accuracy, Interoceptive awareness

## Abstract

Successful social interactions require a good understanding of the emotional states of other people. This information is often not directly communicated but must be inferred. As all emotional experiences are also imbedded in the visceral or interoceptive state of the body (i.e., accelerating heart rate during arousal), successfully inferring the interoceptive states of others may open a window into their emotional state. But how well can people do that? Here, we replicate recent results showing that people can discriminate between the cardiac states (i.e., the resting heartrate) of other people by simply looking at them. We further tested whether the ability to infer the interoceptive states of others depends on one’s own interoceptive abilities. We measured people’s performance in a cardioception task and their self-reported interoceptive accuracy. Whilst neither was directly associated to their ability to infer the heartrate of another person, we found a significant interaction. Specifically, overestimating one’s own interoceptive capacities was associated with a worse performance at inferring the heartrate of others. In contrast, underestimating one’s own interoceptive capacities did not have such influence. This pattern suggests that deficient beliefs about own interoceptive capacities can have detrimental effects on inferring the interoceptive states of other people.

**Supplementary Information:**

The online version contains supplementary material available at 10.1007/s42761-022-00151-4.

Much of our mental life requires us to balance our own mental states (what we feel, what we need) against the mental states of others (what they feel, what they need). Imagine bumping into someone at the grocery isle as you both reach for the last ingredient needed to prepare your favourite dinner. You both quickly retract your hands and face one another. While you already inhabit a particular state (e.g., feeling tired after a long day and wanting to end the interaction without seeming rude), it will further depend on the state of the other person (i.e., do they seem friendly and apologetic or ready to put up a “fight” or simply annoyed). However, unless the person directly informs you of their emotions and desires, you will need to *infer* their emotional and motivational states to adjust your own behavior. The ways in which such inferences occur remain a long-standing question (Adolphs, 2010; Singer & Lamm, 2009; Gallagher & Frith, 2003), but theories of embodied cognition have highlighted the importance of bodily states in providing a window onto the mental states of others (Keysers et al., 2018; Grafton, 2009; Bastiaansen et al., 2009; Goldman & de Vignemont, 2009). This idea relates to the “mirroring mechanism” in action/emotion observation (Ferrari & Rizzolatti, 2014; Gallese & Sinigaglia, 2011; Lamm et al., 2011; Van Overwalle & Baetens, 2009; Wicker et al., 2003), whereby observing actions or bodily states of others may elicit a matching neural representation in the observer’s brain (Gallese, 2003). Such mirroring has been discussed both in the context of our ability to empathize and socially relate to others, and more recent accounts have suggested that one’s own interoceptive abilities are also important for inferring other people’s mental states.

All cognition ensues at the backdrop of continuous visceral signals originating from within the body, like the rhythm of the heart. The neural representation of these ascending signals and their perception is termed interoception (Craig, 2002; Cameron, 2001). While interoceptive signals help regulate body’s homeostatic needs, recent research has demonstrated that interoception can directly shape a range of cognitive processes, including our emotional experiences (e.g., Lange & James, 1922; Craig, 2009; Tsakiris & Critchley, 2016; Feldman et al., 2022). Emotional states may be accompanied by clear changes in facial expressions and actions (e.g., a smile can be associated with the experience of joy), but they can also be accompanied by changes in the viscera (e.g., an angry person may try to keep a straight face, but their heart rate may be accelerating). Whilst no single emotional state is linked to a particular visceral fingerprint (Siegel et al., 2018), meaning that the same visceral change (e.g., accelerating heart) may underpin both anger and fear depending on the context (Hoemann et al., 2020), a perceiver may still use cues related to visceral changes to make inferences about the emotions of others. Notably, recent theoretical frameworks have extended the mirroring-like mechanism to interoception, suggesting that to understand what others might be feeling, we may be simulating (or “mirroring”) their interoceptive states (Ondobaka et al., 2017; Ross & Atkinson, 2020).

For mirror-like mechanism to facilitate action understanding it requires perception of the mirrored actions (i.e., visual input), but also an existing internal motor repertoire (i.e., action knowledge; Calvo-Merino et al., 2005, 2006). Therefore, “mirroring” (or simulating) interoceptive states must also be accompanied by, first, perception of those states in others and, second, a predictive capacity based on one’s own interoceptive experiences. The main challenge with the first requirement (i.e., perception of interoceptive states in others) is that unlike motor behavior, which is perceptually accessible, interoceptive states remain, to a large extent, hidden (Ondobaka et al., 2017). While many changes in the visual appearance happen along the fluctuations in the interoceptive states (e.g., changes in redness of the face, pupil size, head, and eye movements), these are usually very minute (Wu et al., 2012) and rarely explicitly registered by observers. For example, while pupil size can modulate the extent of sadness conveyed by a sad facial expression, participants do not report noticing pupil size changes (Harrison et al., 2007). However, a recent study found that people may be able to infer the heart rate (HR) of others by merely viewing videos of their faces in a neutral emotionless context. Namely, Galvez-Pol et al. (2022) showed participants 10 second videos of two people side-by-side and a square that changed color at the rate of the HR of one of the people. The authors found that observers matched the HR to the correct person above a chance level. They excluded the possibility that the task involved a simple associative learning by showing that replacing actors with images of shapes disrupted the performance to a chance level. In addition, performance degraded when videos were inverted, replaced with still images of the people, and when coloration in the faces was held constant. Thus, perception of at least some interoceptive states, like the HR, may be possible.

The second requirement (i.e., awareness of one’s own interoceptive states) has been more well-researched, but it has not yet been linked to perceiving the interoceptive states in others. While majority of interoceptive processing occurs non-consciously to maintain the physiological condition of the body (i.e., homeostasis), people can become aware of some interoceptive signals to a varying degree. Of the different interoceptive signals that can reach consciousness, individual differences in the ability to consciously perceive one’s heart has been most widely researched. Several tasks have been devised to characterize how well can people objectively perceive cardiac signals (interoceptive accuracy, IAcc; Brener & Ring, 2016). From those the most widely used is the heartbeat counting task (Dale & Anderson, 1978; Schandry, 1981), whereby participants are asked to silently count felt heartbeats without taking their pulse. In addition to objective measures, subjective self-report questionnaires can tap into the extent to which people believe to possess a high IAcc (Garfinkel et al., 2015; Murphy et al., 2019). For example, in the Interoceptive Accuracy Scale (IAS; Murphy et al., 2020), participants are asked to report their beliefs in multiple aspects of interoceptive perception (e.g., “I can always accurately perceive when I am hungry”). While scores on objective and subjective measures can correlate (Murphy et al., 2020), they can also diverge reflecting a poor correspondence between what one believes at the subjective level and what the objective performance shows. This correspondence is sometimes termed interoceptive insight or awareness (Murphy et al., 2019; Khalsa et al., 2018).

The role of interoceptive perception and awareness in emotional processing is well established (Wiens et al., 2000; Wiens, 2005; Pollatos & Schandry, 2008; Barrett et al., 2004). In general, those with better IAcc seem to experience higher levels of arousal (for review see Ainley et al., 2016). More recently, IAcc has been also associated with social cognition (Palmer & Tsakiris, 2018). For example, high subjective IAcc has been associated with improved ability to recognise others’ emotions (Hübner et al., 2021) and objective IAcc predicted higher scores on theory-of-mind items that tapped into making inferences about what another person is feeling, but not what they were thinking (Shah et al., 2017). Furthermore, in a task where participants had to judge the incongruent emotions experienced by oneself and another person (e.g., the participant was viewing a pleasant image, but the other person was listening to an unpleasant sound), participants with higher IAcc were better at both judging the emotion experienced without being biased by the other person and judging what the other person experienced without being biased by one’s own experience (von Mohr et al., 2021). This supports the theoretical position of Palmer and Tsakiris (2018), who argue that better interoceptive perceivers may have a better understanding of how emotional states affect themselves and, in turn, other people. Yet, they can maintain the self-other distinction during social interactions and hence avoid directly sharing other people’s emotional reactions. This is a crucial aspect of higher-level cognitive empathy as opposed to mere emotional contagion or emotion recognition (Stueber, 2018).

However, while the tasks and measures described above tap into relatively high levels of inferring another person’s emotional experiences, Galvez-Pol et al. (2022)’s task tapped into a very basic level of perceiving the interoceptive state of another person, which may, in turn, be one of the building blocks of inferring someone’s affective state (Ondobaka et al., 2017). Therefore, our main research question was whether perception of one’s own interoceptive signals (both objective accuracy, subjective accuracy, and their correspondence) predict the performance on inferring the interoceptive state of another person as measured by the other-HR task developed by Galvez-Pol et al. In addition, given that the ability to infer the internal state of other people may be related to social cognition, we examined whether own interoceptive abilities and other-HR task performance was in turn related to social intelligence. Thus, we employed a pre-registered (https://osf.io/jkwp7) correlational study, whereby each of these capacities was treated as trait-like characteristic. The study was composed of four parts.

First, participants performed the other-HR task (Galvez-Pol et al. 2022), where we aimed to replicate the original findings, whereby participants showed a significantly above chance (50% accuracy) discrimination performance. In addition, to better characterize the other-HR task performance, we asked participants about their confidence, and which cues they relied on while performing the task. Second, participants performed the heartbeat counting task across three randomly intermixed time intervals (25 s, 35 s, 45 s), which characterized their objective interoceptive perceptual abilities (objective IAcc). Notably, the task was conducted online, and we used a photoplethysmography algorithm (rPPG) on participants’ webcam recordings to estimate their true HR (van der Kooij & Naber, 2019). The rPPG was used to calculate the HR by estimating the changes in the blood flow on the skin. Importantly, HR estimates from this rPPG method (from individual webcam recordings via a remote online setting as here) were comparable to those obtained from a validated mobile heart rate application (Di Lernia et al., 2022). Third, participants completed the Interoceptive Accuracy Scale (IAS; Murphy et al., 2020), which assessed their subjective self-reported IAcc. Finally, participants also completed the Tromsø Social Intelligence scale (SI; Silvera et al., 2001; Grieve & Mahar, 2013), which characterizes participants’ self-perceived social competence in terms of social information processing (i.e., how well they believe they can process social information), social awareness (i.e., how aware they are of social cues and reactions), and social skills (i.e., how skilful they believe themselves to be in social situations). The scores on SI have been associated with scores on the Empathy Quotient (Lawrence et al., 2004), which taps into cognitive empathy, emotional reactivity, and social skills (Grieve & Mahar, 2013). The scores on social processing subscale have been linked to greater experience of empathetic concern and enhanced ability to take others’ perspective (Grieve & Panebianco, 2013) as well as higher rate of spontaneous mimicry (Genschow et al., 2018). Thus, it seems that SI scores are related to both the cognitive and the affective aspects of empathy.

## Method

### Participants

The data was collected via an online participant pool—Prolific (https://app.prolific.co/). The study was approved by Royal Holloway’s Ethics Committee and signed informed consent was obtained from all participants. The sample size estimation was based on detecting a relationship between objective IAcc and other-HR task performance. Although no previous study has examined this relationship, Shah et al. (2017) found a significant correlation between IAcc and a task that required representation of other’s emotions (*r* = .41, *p* < .001, *n* = 72). This means that if we were to run a correlation between IAcc and the other-HR task, we needed 43 participants to reach a power of .80 (G*Power; Faul et al., 2007). For a regression analysis, in an unpublished dataset, exploratory analyses revealed a significant effect of IAcc on externally oriented thinking subscale of Toronto Alexithymia Scale (Leising et al., 2009), which refers to a lack of emotional engagement, *t*(66) = −2.192, *p* = .03, multiple *R*^2^ = .068. For a model with a single predictor, a sample size of 87 participants should reach power of .80. However, online experiments decrease performance and increase variance. Thus, we aimed for a sample of 100 participants.

Participants had to be between 18 and 39 years of age and be fluent in English. They had to have access to a computer or a laptop with a webcam, good internet connection, and run the experiment in Google Chrome browser. Eligible participants had to correctly fill a quiz on experimental requirements: (1) whether they can perform the experiment on a mobile phone (correct answer: no); (2) whether they need a computer/laptop with a webcam (yes); (3) whether they must not open other tabs during the experiment (yes); (4) whether they must sit still during the webcam recordings (yes); (5) whether they can perform the experiment at night in a dark room (no). Incorrect answer to any of these questions automatically prevented the person from participating.

Further participants’ data was excluded in two steps. First, we excluded those who failed at least one of the attention checks throughout the experiment. These included (1) during the other-HR task, in two trials, instead of videos, participants were presented with cartoon images of owls scattered across the screen. They had 20 s to insert the number of presented owls (5 and 7); (2) in each questionnaire, one item told participants to pick a specific response (agree and disagree); (3) in the Interoceptive Accuracy Scale (IAS), we asked participants to specify what they thought the term ‘accuracy’ meant in the questionnaire context, where they could have chosen either: a) “How much attention you pay to these sensations” (incorrect); b) “How accurate you are at perceiving these sensations” (correct); c) “How often or how intensely these sensations actually occur in your body” (incorrect). Thirty participants were excluded due to failure at these checks. Second, remaining participants whose rPPG data was insufficient to provide reliable heart rate (HR) measure were not used for the objective interoceptive performance analysis. Forty-seven participants failed the criteria for a reliable rPPG data, because either (a) they were missing at least one of the recordings due to technical issues; (b) rPPG algorithm did not detect their face in at least one of the intervals; (c) the video quality was too poor to provide a reliable rPPG (i.e., frame rate was below 20; Di Lernia et al., 2022); or (d) the variability between detected HR across sequential intervals exceeded 10 bpm, suggesting unreliable readings.

Because some participants passed the attention checks, yet their videos were unsuitable for extracting their HR, we did not wish to discard their data completely. Thus, we performed analyses in two steps. In step 1, we included all individuals who passed the attention checks (*N* = 143; 65 women, mean age = 25.5, mean BMI = 24.0 ± 4.3 SD) examining their performance on the other-HR task and its relationship to self-reported social intelligence and subjective interoceptive abilities, independently of their IAcc performance. In step 2, we only included in the analysis the individuals whose videos were of sufficient quality to reliably detect their HR via rPPG (*N* = 96; 45 women, mean age = 28.8, mean BMI = 23.7 ± 3.8 SD) allowing us to examine their performance on the other-HR task and its relationship to objective interoceptive ability. Data collection was continued in chunks of 20 participants until the 2^nd^ dataset (*N* = 96) contained approximately 100 reliable participants.

### Design and Procedure

The whole experiment was conducted via an online experiment builder—Gorilla (https://app.gorilla.sc/). First, participants performed the other-HR task replicated from Galvez-Pol et al. (2022; see the original paper for full methods). They were presented with 10-s videos composed of two actors, side-by-side, with a square between the videos changing color from red to black in the rate of the heart rate (HR) of one of the actors. The actors were filmed from the shoulders to the top of the head, looking straight towards the camera, remaining still, with a neutral expression and direct gaze. Video recordings were accompanied by simultaneous electrocardiogram (ECG) recording of actors’ HR—this was used to create the square that conveying cardiac feedback. Each actor (*n* = 7) had 5 different 10-s shots randomly selected from a total of 2-min recording. Resulting 35 videos were then combined via permutation to create 84 video combinations (order of video combinations randomized across participants). In half of the videos, the square represented the HR of the actor on the right side of the video, while in the other half it belonged to the left actor. Participants, after watching the 10-s video combination, were asked to indicate to which actor the HR belonged to, by pressing A, if it belonged to the actor on the left, or pressing K, if it belonged to the actor on the right. After entering their response, participants were asked to indicate their confidence on a scale from 0 (not confident at all) to 10 (absolutely confident). The order of videos was randomized for each participant. At the end of the task, we asked participants: “*Could you tell us which cues from the videos did you use to perform this task*?”. The task took approximately 30 min.

Then, participants performed the heartbeat counting task, whereby they were asked to silently count felt heartbeats without taking the pulse over three different time intervals (25 s, 35 s, 45 s—order of intervals counterbalanced across participants) and report the number of beats they counted. During the counting intervals, participants’ face was recorded via their webcam, which allowed the offline estimation of their true HR via a photoplethysmography (rPPG) technique that measured changes in the blood flow on the skin. To make sure that participants were not using their pre-existing knowledge of their HR to perform the heartbeat counting task (Ring & Brener, 1996; Murphy et al., 2018), we asked participants what they believed their resting state HR to be. We expect the knowledge of HR (accuracy at which participants report resting state HR) to be unrelated to accuracy at HR counting. This task took approximately 5 min.

Lastly, participants filled two Likert-style questionnaires. Subjective interoceptive accuracy was measured with the Interoceptive Accuracy Scale (IAS; Murphy et al., 2020), whereby participants indicated how much they agreed or disagreed with 21 statements concerning the accuracy at which they can perceive their interoceptive signals (5-point scale: strongly disagree, disagree, neither agree nor disagree, agree, strongly agree). The exact items can be seen in supplemental Figure S1.1. The resulting score can range from 21 (low subjective accuracy) to 105 (high subjective accuracy). Perceived social intelligence was measures with a 21-item Tromsø Social Intelligence questionnaire (SI; Silvera et al., 2001), whereby participants have to indicate on a 5-point scale how much they agree or disagree with statements about: (a) how well they believe they can process social information (social information processing; SP), (b) how aware they are of social cues and reactions (social awareness; SA), and (c) how skilful they believe themselves to be in social situations (social skills; SS). Each subscale consisted of 7 items (see supplemental Table S1). The scores on each subscale could range from 7 to 35. The order of the two questionnaires was counterbalanced across participants and the order of items within each questionnaire was randomized. The questionnaires took approximately 5 min to complete.

### Statistical Analysis

The analysis plan was pre-registered prior to the data collection (https://osf.io/jkwp7), but some analysis steps were omitted, while others were explored in more depth. As stated in the pre-registration, the replication of other-HR task involved calculating the mean proportion of correct response for each participant and submitting these to a one-sample *t*-test against 0.5 (chance level). We also used signal detection theory to distinguish between other-HR bias-free discriminability (d prime) and choice bias (i.e., propensity to choosing left or right actor). Discriminability values above 0 indicate increasingly stronger ability to discriminate between the owner of the heart rate (HR), while choice bias values close to 0 indicate unbiased performance (Macmillan & Creelman, 2004). However, because d prime values were almost perfectly correlated with accuracy, and no considerable choice bias was evident, we did not analyze those measures further. We did, however, use choice bias to analyze whether any of the actors biased the performance on the other-HR task as explained in the pre-registration. This analysis is shown in the supplemental material (see section S2).

Another change in terms of preregistration is that we could not calculate the metacognitive sensitivity (type-2 signal detection metric or meta d prime), because there were not enough trials for each confidence level even when we reduced our confidence scale to three levels to successfully distinguish between the two response choices. Therefore, we characterized metacognitive awareness by looking at the difference in confidence rating between correct and incorrect responses. If this difference was statistically significant, it meant that participants possessed some metacognitive awareness of their performance. That is, their confidence tracked the correctness of their response.

At the end of the study, we asked participants to describe the cues they used to perform the other-HR task (“*Could you tell us which cues from the videos did you use to perform this task*?”). We then tried to categorize the prompts into discrete 8 categories (Fig. 2). This part was not pre-registered and provided exploratory qualitative extension to our findings.

Objective interoceptive accuracy (IAcc) was calculated from the heartbeat counting task as the difference between true and reported heart beats per interval following the Eq. 1 (Schandry, 1981):

1 $$ IAcc=\frac{1}{3}\sum 1-\frac{\left|{HB}_{real}-{HB}_{count}\right|}{HB_{real}} $$

where resulting scores take on values from 0 to 1 – 1 depicting perfect accuracy. Subjective interoceptive accuracy and social intelligence were derived from the questionnaires described above. As stated in pre-registration, we also estimated interoceptive awareness as the match between confidence in heartbeat counting accuracy and actual accuracy. Because we only had three heartbeat counting trials, we could not estimate the correlations coefficient between the two (Garfinkel et al., 2015). Instead, we first transformed confidence ratings from scale 0 to 10 to values ranging from 0 to 1 and then for each interval we took the difference between IAcc and confidence rating, which was then averaged to get a mean measure of interoceptive awareness, as shown in Eq. 2:

2 $$ IAwareness=\frac{1}{3}\sum I{Acc}_{each\ interval}- Confidence $$

In addition, we also estimated interoceptive insight (Garfinkel et al., 2015), which is believed to reflect how well self-reported interoceptive beliefs (as measured by IAS in the present study) track objective IAcc. For this, we first transformed total Interoceptive Accuracy Scale (IAS) scores that ranged from 21 (low beliefs) to 105 (high beliefs) to ranging from 0 to 1 and calculated the difference between IAS and mean IAcc, as shown in Eq. 3:

3 $$ IInsight= IAcc-{IAS}_{standardized} $$

Before the main analysis, we wished to ensure that the HR and the IAcc estimated through the rPPG algorithm ran on webcam videos reflected participants’ true performance rather than noise from video recordings. First, we looked at the consistency of HR and IAcc across the three heartbeat counting trials (25s, 35s, 45s). Second, 61 participants from the study also performed another session of heartbeat counting task only. This allowed us to check the consistency of the objective interoceptive performance across two different sessions. To check the consistency, we employed one-way repeated-measures ANOVAs or paired-sample *t*-tests to ensure no statistically significant differences between the measures. In addition, we estimated test-retest reliability with intra-class correlation coefficient (ICC; Shrout & Fleiss, 1979; Koo & Li, 2016), which was computed with ICC function of “psych” package (Revelle, 2021) in R.

Finally, as already described in the participants section, we first explored the correlations between other-HR task accuracy and subjective measures on a bigger sample (*N* = 143). Due to the stringent exclusion criteria for rPPG processing, IAcc could be estimated in 96 participants. Thus, we ran our linear regression model on the reduced sample with IAcc. Because we did not find any direct influence of IAcc on other-HR performance, we omitted the mediation analysis described in the pre-registration. Instead, we looked at beta estimates for each of our measures (IAcc, IAS, and self-perceived social intelligence) and their interaction, while controlling for influences from gender, BMI, average HR, and the difference between true and estimated resting HR as control variables. For the linear regressions, assumptions of linearity, normality, and homoskedasticity were visually evaluated from the model residual plots. Significant interactions were followed up by simple slopes analysis (Bauer & Curran, 2005) by looking at the effect of one continuous variable when the interacting continuous variable was either low (-1 SD from the mean), at its mean value, and high (+1 SD from the mean). We used sim_slopes function from the “interactions” package in R (Long, 2019).

## Results

### People Can Infer the Heart Rate of Other People (n = 143)

**Fig. 1:**
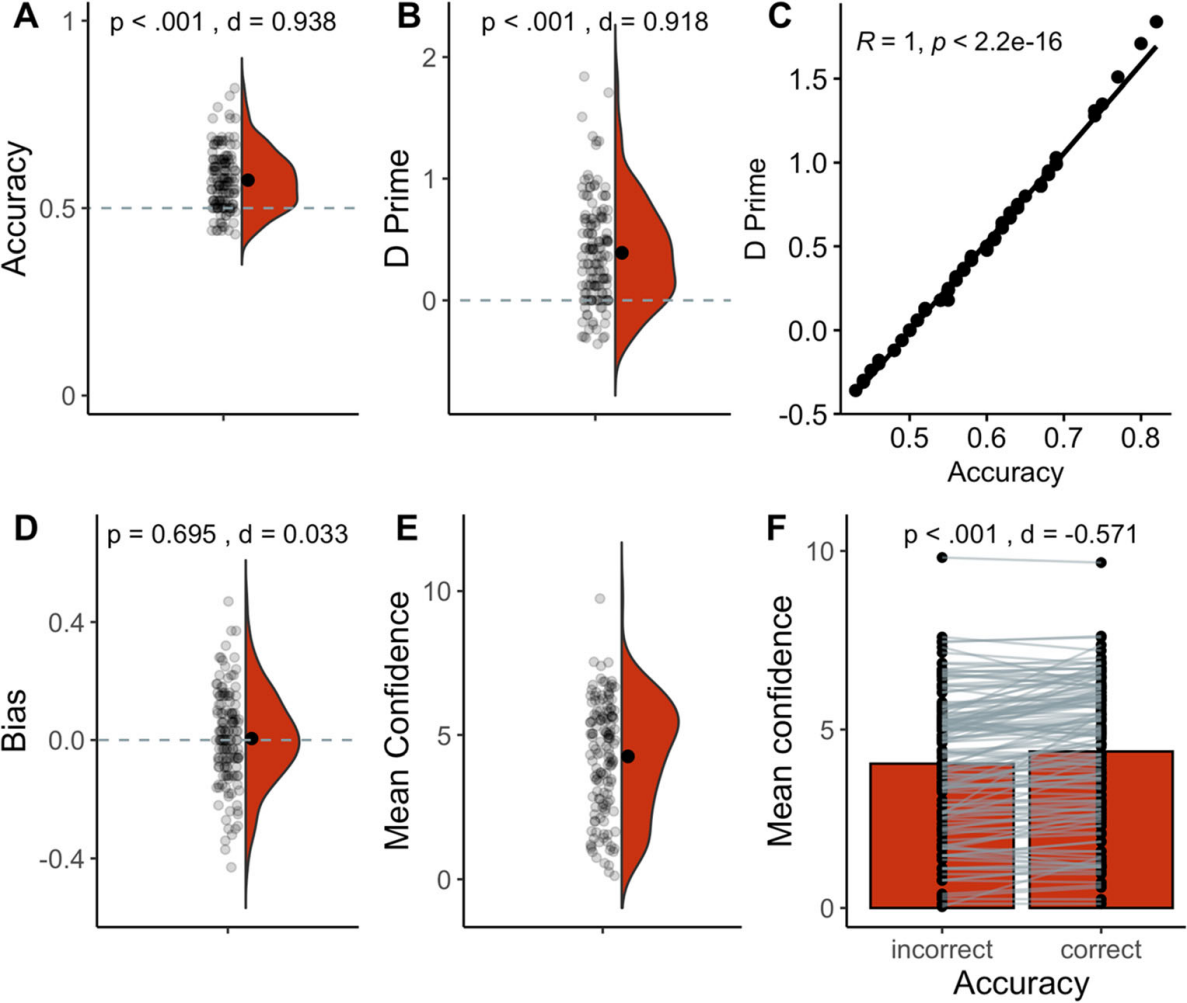
Summary of performance on other-HR task (*n* = 143). **A** Accuracy as the proportion of correct responses. Statistics refer to one-sample *t*-test against chance level (0.5). **B** D prime reflects bias-free discriminability (i.e., accuracy independent of propensity to choose left or right response). Statistics refer to one-sample *t*-test against chance level (0). **C** Correlation between accuracy and d prime. **D** Bias or the propensity of choosing left or right response. Statistics refer to one-sample *t*-test against 0 (absence of a bias). **E** Mean confidence rating. **F** Mean confidence as a function of incorrect or correct response. Statistics refer to paired-sample *t*-test

The performance on the other-HR task was significantly above chance level both when considering proportion of correct responses; one-sample *t*-test against 0.5: *t*(142) = 11.2, *p* < .001, *d* = .94; Fig. 1A and bias-free d prime, one-sample *t*-test against 0: *t*(142) = 11.0, *p* < .001, *d* = .92; Fig. 1B. The two measures were strongly correlated (*r* = 1, *p* < .001; Fig. 1C) and there was no evidence of left- or right-response bias, one-sample *t*-test against 0: *t*(142) = 0.4, *p* = .69, *d* = .03; Fig. 1D. Mean accuracy was 0.6 (SD = 0.08) with scores ranging from 0.4 to 0.8 (Fig. 1A). Mean confidence was 4.3 (SD = 1.9). Accuracy and reported confidence did not correlate (*r* = .06, *p* = .46), meaning that participants who performed well on the task did not necessarily report high confidence overall. However, Fig. 1F shows that participants reported higher confidence, on average, following correct responses (mean = 4.4, SD = 2.0) than following incorrect responses, mean = 4.1, SD = 1.9; paired-sample *t*-test: *t*(142) = −6.8, *p* < .001, *d* = −.57. Indeed, the difference in confidence between correct vs. incorrect responses was positively correlated with accuracy (*r* = .44, *p* < .001), meaning that better performance was accompanied by better metacognitive awareness. In sum, we replicated the finding of Galvez-Pol et al. (2022)—people can infer the HR of others in an online experimental context above a chance level. In addition, while better perceivers did not necessarily report high confidence, their confidence ratings did distinguish correct from incorrect responses, indicating some metacognitive awareness.

### What Cues Participants Used to Discriminate Actors’ Heart Rates?

**Fig. 2:**
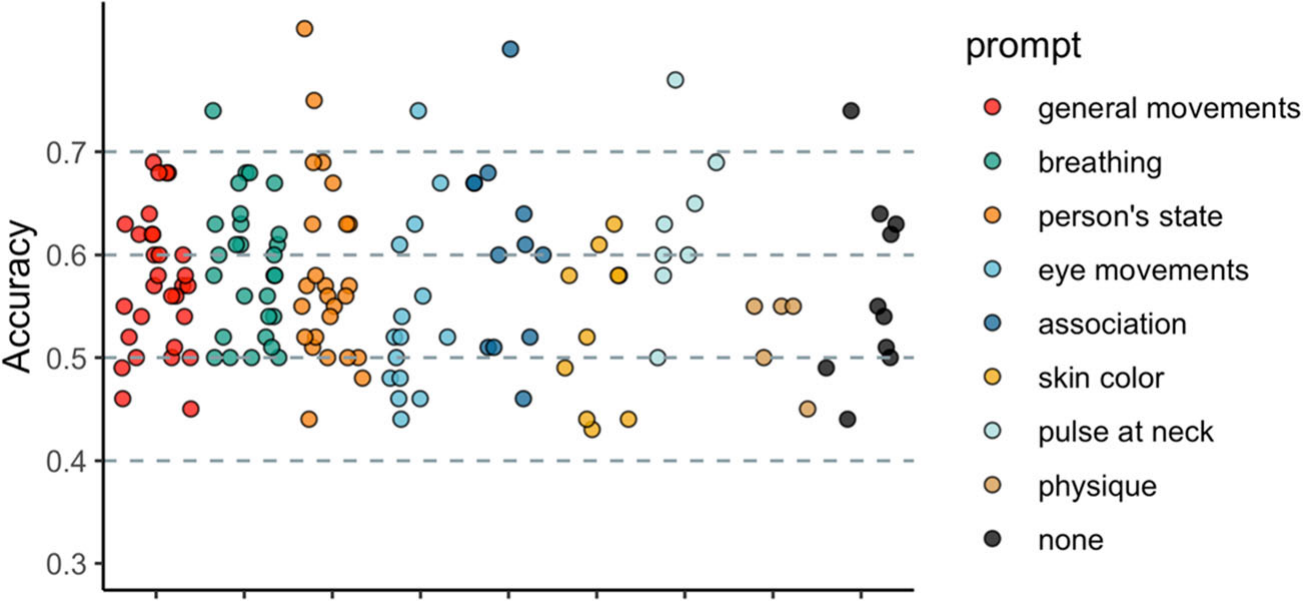
Task performance categorized based on the prompts participants used to describe which cues they used to perform the task. The prompts were divided into 8 categories (shown in ascending order from the most popular to the least)

At the end of the task, we asked participants: “Could you tell us which cues from the videos did you use to perform this task?” 133 out of 143 participants provided a clear answer. We tried to categorize the prompts into discrete 8 categories (Fig. 2). Often participants mentioned more than one factor, for example, when mentioning skin color, a large number also mentioned general body movements. In these cases, we categorized the prompts based on the most distinctive feature. Section S3 of supplementary material shows how many times each prompt was mentioned in total, even if by the same participant.

1. General movement (*n* = 30): this category includes participants who did not emphasize a particular movement type like eyes or breath, but either referred to both or mentioned multiple movement cues (e.g., eyes, breath, swallowing, head, and body movements), e.g., “I looked at eye movement and body language, also i tried to look at their breathing,” “Movement, breathing, blinking and even the swallowing of saliva.”
2. Breathing (*n* = 28): participants specifically referred to breathing and breathing movements (e.g., rising of the chest/shoulders) to help them determine the heart rate, e.g., “I was observing the way the person was breathing,” “In the beginning, I was paying attention to the faces and whether I could see their nostrils moving with their breaths, then I started looking at the movement of their shoulders.”
3. Person’s state (*n* = 25): participants emphasized how the actors looked to them, whether they looked nervous or calm, this category also includes references to facial expression, e.g., “Mainly how anxious I thought the person looked, but also other physical cues (blinking, swallowing, etc),” “I tried to look at how uncomfortable or agitated the person looked, so who swallowed more, whose eyes were darting round, who seemed most comfortable and at ease, then I used these judgments to decide who the heartbeat belonged to.”
4. Eye movement and blinking (*n* = 15): participants specifically mentioned looking for eye movements and blinking, some participants also mentioned swallowing, e.g., “The frequency of eye blinking, swallowing and looking at the other side,” “I tried to reach my conclusion by seeing how many times they closed their eyes, if they looked somewhere else and their body movements.”
5. Association (*n* = 12): participants specifically mentioned forming an association between specific heart rate and specific actor, e.g., “I tried to see use comparison between heart rates and certain people to try and figure out which person generally has what heart rate.,” “The rate at which the block flashes in regards to different people. Sometimes I could see when person A for example is involved in one comparison with person B then another comparison with person C, and then person B and C, you could kind of pick up who beats faster and slower.”
6. Skin color (*n* = 10): participants mentioned skin color as a cue they used, sometimes it was mentioned along general movements, but if the prompt included color, it was categories into this category, e.g., “In the first half I thought about their movements but in the second half I started noticing also slight changes in the color of their faces (blushing and such),” “The skin color and maybe how much healthier seems to be.”
7. None (*n* = 10): these participants did not provide any prompts, or their prompts were unclear, but some of them referred to general intuition.
8. Pulse at the neck (*n* = 8): participants mentioned looking at the neck to estimate the pulse of the actors, e.g., “I was mainly looking at neck to see if I can feel the pulse,” “throbbing in the neck.”
9. Physique (*n* = 5): some participants specifically mentioned the appearance and fitness of the participants, e.g., “The size of face. If they are male or female. If they are skinny or not,” “At first I think my intuition was driven mostly by the rate of eye blinking but as the test went on I started to correlate the heart beat more to the differences in body type.”

### Inferring Others’ Heart Rates Does Not Correlate with Subjective Interoceptive Accuracy or Social Intelligence

Before examining the objective interoceptive performance on the reduced dataset (due to rPPG exclusion criteria), we examined the relationship between other-HR task performance and subjective interoceptive accuracy (as measured by the Interoceptive Accuracy Scale, IAS) as well as self-reported social intelligence (as measured by the Social Intelligence Scale, SI). The mean score (mean total score = 81) and the standard deviation (SD = 8.7) of IAS were similar to those reported in Murphy et al. (2020; Study 5), ranging from 53 to 104 (see supplemental Figure S1.1). Scores on SI subscales (see supplemental Figure S1.2) were lower than those reported in Silvera et al. (2001; Study 3). The mean total score was 57.8 (SD = 6.8) ranging from 35 to 69. The subscales of the SI were not all correlated (see supplemental Figure S1.2). Specifically, scores on the subscale of social awareness were not correlated with scores on social processing (*r* = −0.1, *p* = .24) or social skill (*r* = 0.1, *p* = .38), but they were all correlated with the total score. Thus, we used the total score to index the self-reported social intelligence.

Table 1 shows correlations between performance measures on the other-HR task (accuracy, mean confidence, difference in confidence between correct vs. incorrect responses) and the questionnaire scores reflecting subjective interoceptive accuracy (IAS) and self-perceived social intelligence (SI). The only significant correlation is a positive association between confidence rating on other-HR task and IAS score, but such correlation may simply reflect a general propensity to choose extreme sides of the scale. Neither other-HR task performance nor metacognitive awareness shared a statistically significant association with subjective interoceptive accuracy nor with social intelligence. We next examined the objective interoceptive performance.

**Table 1:** Correlations between other-HR task performance and questionnaire scores

Other-HR task	IAS	SI
Accuracy	−.08	−.04
Confidence	.26**	.17
Difference in conf. between correct-incorrect responses	−.05	−.04

*Note*: *p < .05, **p < .01; *N* = 143; *IAS* Interoceptive Accuracy Scale; *SI* Social Intelligence Scale

### Objective Interoceptive Accuracy Can Be Consistently Obtained from Online rPPG (*n* = 96)

**Fig. 3:**
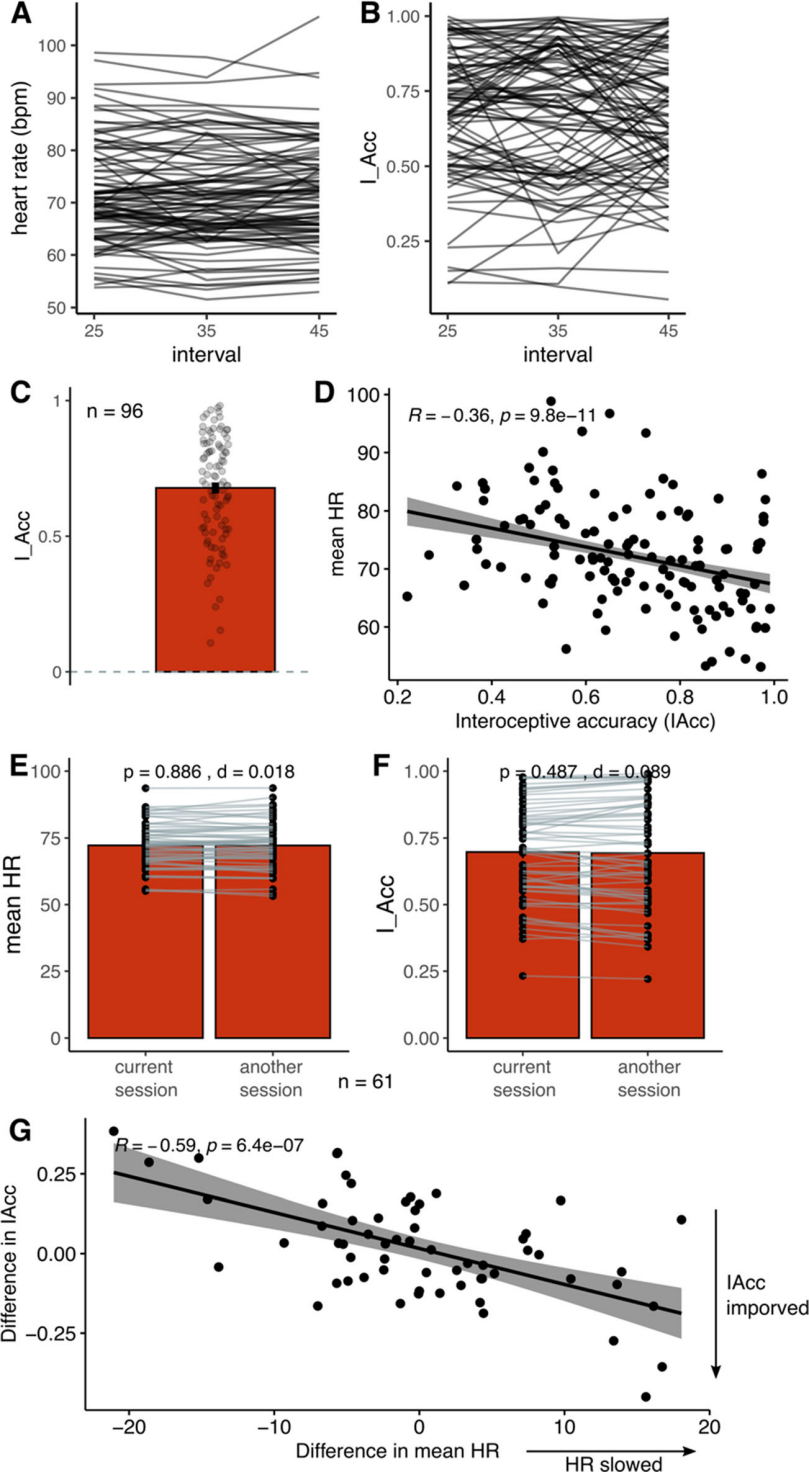
Objective interoceptive ability estimated as the difference between true and counted heartbeats across three intermixed intervals (25 s, 35 s, 45 s). **A** Heart rate across the intervals for each participant (note that in the experiment the order of intervals was random). **B** Interoceptive accuracy at each interval for each participant. **C** Mean interoceptive accuracy (values can range between 0 no accuracy to 1 perfect accuracy). **D** Relationship between accuracy and average heartrate. **E** 61 participants from the study also performed another session of heartbeat-counting task only. There was no significant difference between mean heartrates across the two sessions. **F** There was no significant difference between heartbeat counting accuracy across the two sessions. **G** Relationship between the change in the interoceptive accuracy and the change in average HR across the two sessions. There was a significant negative relationship, meaning that as the heart rate slowed, interoceptive accuracy improved

Before looking at the predicted relationship between own-HR and other-HR perceptual abilities, we wanted to ensure that the heart rate (HR) that was estimated through the rPPG algorithm ran on webcam videos reflected participants’ true HR rather than noise from video recordings. This is the reason why stringent exclusion criteria were applied to ensure that only videos of sufficient quality were included. Figure 3A, B shows the variability in HR and interoceptive accuracy (IAcc) between the heartbeat counting trials (25 s, 35 s, 45 s). There were no significant difference between the intervals both in terms of HR, one-way ANOVA: *F*(1,95) = 0.01, *p* = .95) and in IAcc, *F*(1,95) = 1.41, *p* = .24. In addition, test-retest reliability, estimated via intra-class correlation coefficient (ICC), was strong for the HR (ICC = .93, *p* < .001, 95%CI: .91 to .95) and moderate but significant for IAcc (ICC = .74, *p* < .001, 95%IC: .68 to .80). Note that ICC of 0.6 are what is normally observed for behavioral tasks, ICC is typically much higher for self-report measures (e.g., Enkavi et al., 2019; Clark et al., 2022). Mean IAcc estimated as the difference between true and counted heartbeats across three intermixed intervals (25 s, 35 s, 45 s) was 0.68 (SD = 0.11) ranging from 0.20 to 0.98 (Fig. 3C). This is comparable to previous studies that have used ECG instead of rPPG (e.g., Garfinkel et al., 2015; Ainley et al., 2020).

IAcc was negatively correlated with mean HR, meaning that people with slower heart rates produced more accurate heartbeat counting scores (Fig. 3D). This is a common finding (Ainley et al., 2020), which may arise because it is easier to attend to slower heartbeats, but also simply due to the IAcc calculation, where greater denominator (slower HR) would lead to smaller IAcc. We did not ask participants to count seconds as a control task, but previous studies generally fail to find that participants who show high accuracy at counting seconds necessarily also perform well on counting heartbeats (e.g., Murphy et al., 2020). We did ask participants to report their believed resting state HR. Notably, IAcc did not share a significant correlation with the difference between average and estimated resting state HR (*r* = −0.17, *p* = 0.1), indicating that participants were unlikely to rely on their knowledge of their heart rate to accurately perform the task.

Sixty-one participants from the study also performed another session of heartbeat counting task only. This allowed us to check the consistency of the IAcc. Figure 3E, F shows that both mean HR and mean IAcc tended to stay consistent across the two sessions (paired-sample t-test *p* values: .89 and .49, respectively). In addition, test-retest reliability, estimated via intra-class correlation coefficient (ICC), was moderate but significant for both the mean HR values (ICC = .60, *p* < .001, 95%CI: .45 to .72) and IAcc (ICC = .64, *p* < .001, 95%IC: .50 to .75). Figure 3G indicates that participants whose IAcc improved between sessions were associated with decrease in HR.

Furthermore, we examined the relationships between the interoceptive measures. Table 2 shows correlation coefficients across objective interoceptive accuracy (accuracy on heartbeat counting), mean response confidence (on heartbeat counting), interoceptive awareness (accuracy—confidence on heartbeat counting), total score on the subjective accuracy scale (IAS), interoceptive insight (accuracy on heartbeat counting—IAS score). First, objective interoceptive accuracy (IAcc) was weakly correlated with mean confidence in the heartbeat counting (*r* = .23, *p* = .02), indicating that higher mean accuracy was related with higher mean confidence. IAcc was uncorrelated with IAS score (*r* = .08, *p* = .45) indicating that those with high IAcc did not necessarily report high beliefs in their interoceptive abilities. Interestingly, IAcc was strongly correlated with both awareness (IAcc—confidence) and insight (IAcc—IAS). In both cases, higher IAcc was associated with more positive values on awareness and insight, indicating a tendency to underestimate one’s own interoceptive abilities. Note that mean awareness across participants was 0.13 (SD = 0.26) and insight was −0.04 (SD = 0.21).

**Table 2:** Correlations between interoceptive measures

	mConfidence	IAcc—Conf	IAS	IAcc—IAS
IAcc	.23*	.58***	.08	.90***
IAS	.25*	−.15		−.37***

*Note*: *p < .05, **p < .01, ***p <.001; *N* = 96; *IAcc* interoceptive accuracy; *IAS* Interoceptive Accuracy Scale; *mConfidence* mean confidence across all heartbeat counting trials; *IAcc—Conf* interoceptive awareness; *IAcc—IAS* interoceptive insight

### Own Interoceptive Abilities Alone Do Not Predict Performance in Guessing Others’ Heart Rates, but Inflated Interoceptive Beliefs Have a Negative Effect (*n* = 96)

Table 3 shows the correlations between all the relevant variables in the experiment. All correlations that reached statistical significance remained weak (*r* ~ .20). Scores on the subjective questionnaires (IAS & SI) shared a significant positive correlation (*p* = .04), but interoceptive measures of awareness and insight shared a significant negative correlation with scores on SI (*p* = .04 and .03, respectively). This means that underestimation of interoceptive abilities was related to lower self-perceived social intelligence, which may reflect a general propensity to underestimate subjective measures. When looking at the associations with the other-HR task performance both in terms of accuracy and metacognitive awareness, the only significant, but weak, correlation emerged between interoceptive metacognitive awareness (IAcc—confidence) and metacognitive awareness on the other-HR task (*p* = .03). This suggests that people used confidence ratings to estimate their performance similarly across the two tasks.

**Table 3:** Correlations between all the relevant variables in the experiment

	SI	Other-HR accuracy	Other-HR metacognitive awareness
IAcc	−.13	.11	.10
IAS	.21*	−.05	.01
IAcc—confidence	−.21*	.15	.23*
IAcc—IAS	−.22*	.13	.09
SI		−.07	.03

*Note*: *p < .05; *N* = 96; *SI* Social Intelligence Scale; *IAcc* interoceptive accuracy; *IAS* Interoceptive Accuracy Scale; *IAcc—Conf* interoceptive awareness; *IAcc—IAS* interoceptive insight; *Other-HR metacognitive awareness* difference in confidence between correct and incorrect responses

**Fig. 4:**
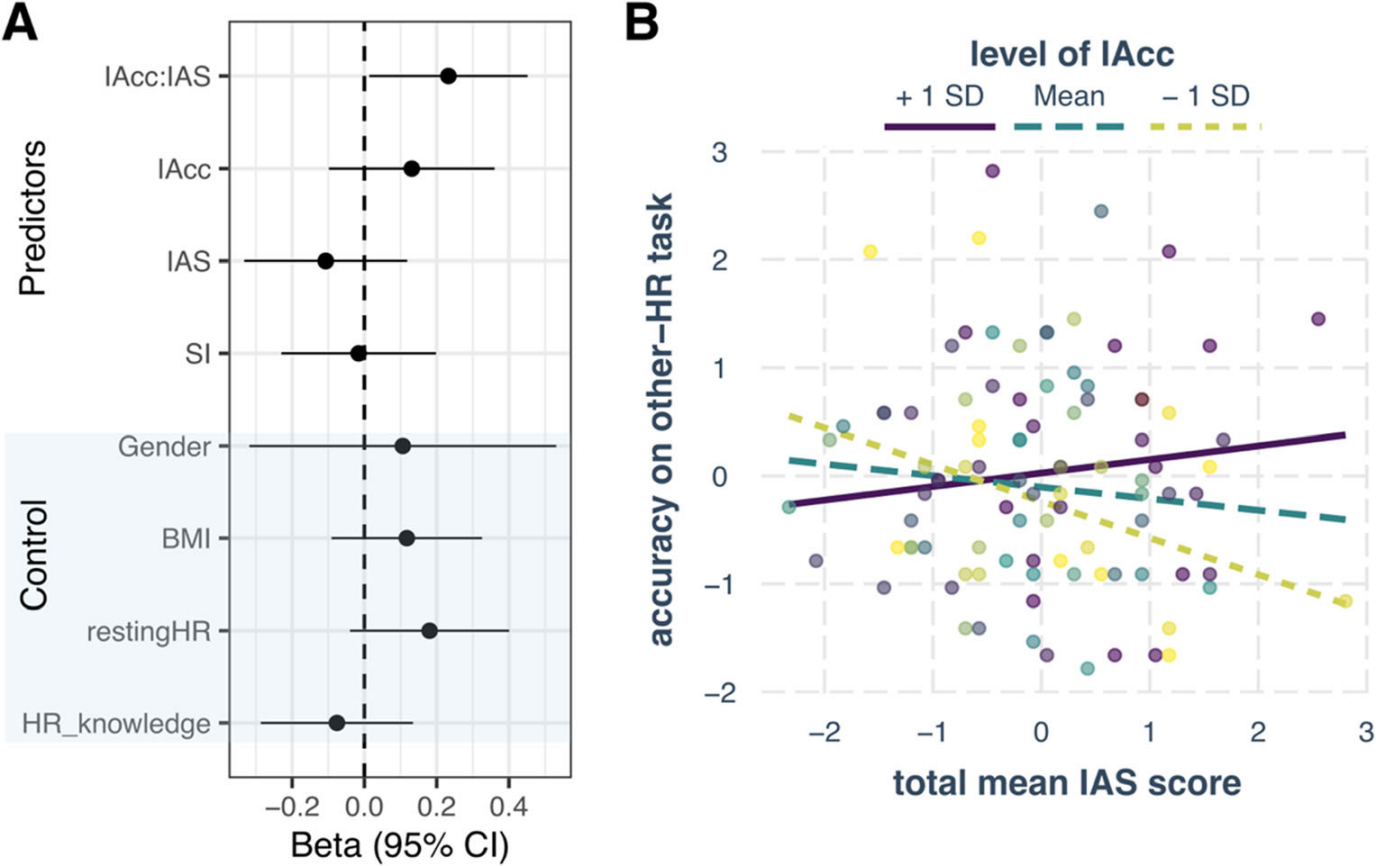
Predicting accuracy in the other-HR task, where participants were inferring the heart rate of another person. **A** Beta estimates and 95% confidence intervals for the predictors and the control variables. The only significant contribution came from the interaction between objective and subjective interoceptive measures. **B** the interaction was broken down with simple slopes analysis by looking at the contribution of IAS on other-HR task accuracy between high objective heartbeat counting accuracy, IAcc (purple), mean IAcc (green), and low IAcc (yellow). The effect of IAS predicted lower other-HR accuracy when IAcc was low (*p* = .04)

Next, although the exploratory correlations did not reveal the predicted relationships with the ability to infer the heart rate of other people, we ran the pre-registered regression analysis with mean-centered and standardized variables: IAcc, IAS score, SI score, and their interactions. We added gender (0 for male, 1 for female), BMI, average HR, and the difference between true and estimated resting HR as control variables. We did not add interoceptive awareness (IAcc—confidence) nor insight (IAcc—IAS) to the regression, as these shared strong correlations with IAcc. Assumptions (linearity, normality, homogeneity of variance) were checked, and no clear violations were identified. The model accounted for 13% of variance (multiple *R*^2^ = 0.13) and the only statistically significant contribution was from the interaction between objective and subjective interoceptive accuracy (beta = 0.23, *p* = .04; Fig. 4A). Metacognitive awareness in the other-HR task performance (difference in confidence between correct-incorrect responses) was not predicted by any of the predictors or their interactions.

The significant interaction between IAcc and IAS scores was followed up by simple slopes analysis (Bauer & Curran, 2005) by looking at the effect of IAS on the accuracy in other-HR task at three levels of IAcc: when IAcc is low (−1 SD from the mean), when IAcc is at its mean value, and when IAcc is high (+1 SD from the mean; see Fig. 4B). The effect of IAS on other-HR accuracy was significant only at the low level of IAcc (beta = −0.34, *p* = .04), but not at the mean level of IAcc (beta = −0.11, *p* = .35) or the high level of IAcc (beta = 0.13, *p* = .42). This means weaker ability to perceive one’s own heartbeats (low IAcc) and heightened subjective interoceptive beliefs (e.g., “I can always accurately perceive when my heart is beating fast”) was predictive of worse performance at guessing the HR of observed actors.

## Discussion

**Fig. 5:**
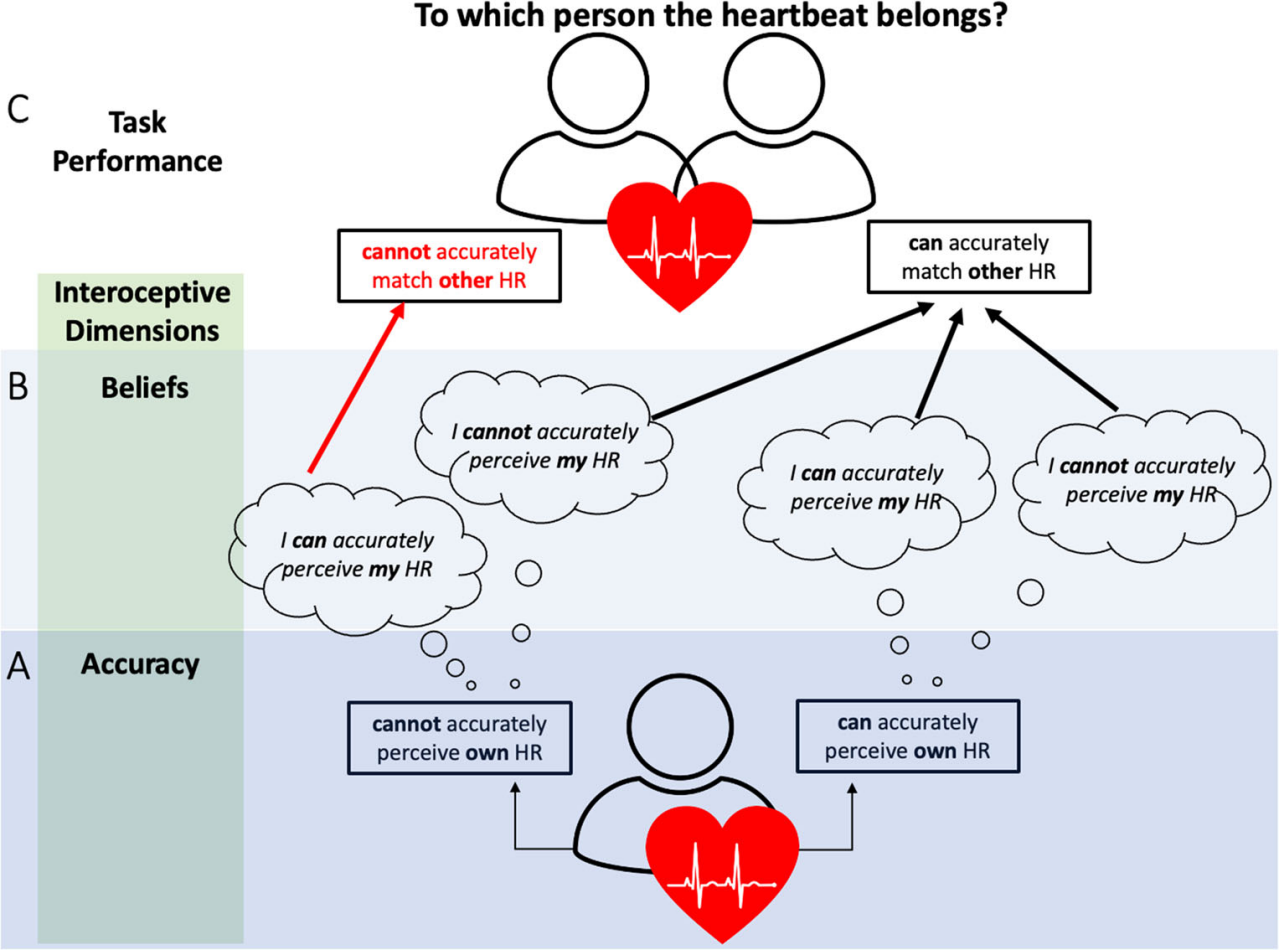
Relationship between (**A**) the objective accuracy in tracking one’s own cardiac signals as measured with heartbeat counting task and (**B**) the self-reported beliefs about accuracy in perceiving own interoceptive signals as measured with Interoceptive Accuracy Scale (IAS) with (**C**) the accuracy in matching a heart rate to its owner (other-HR task). Neither objective interoceptive accuracy or IAS directly predicted other-HR performance. However, when objective interoceptive accuracy was low, IAS scores predicted other-HR accuracy. Specifically, those who falsely believed to be good interoceptive perceivers performed worse on other-HR task, whereas those who accurately believed themselves to be worse interoceptive perceivers performed better. This was not the case for those who were already objectively good interoceptive perceivers. This suggests that overestimating one’s own interoceptive abilities can have detrimental effects on inferring interoceptive states of others

Inferring the interoceptive state of another person may open a window into their feeling states (Ondobaka et al., 2017). Yet, unlike actions and facial expressions that can be easily perceived, interoceptive states tend to remain hidden from direct perception. Nonetheless, Galvez-Pol et al. (2022) observed that people can match a heart rate to its owner with varying accuracy (other-HR task). Here, we replicate these findings and show that high accuracy is accompanied with a higher metacognitive awareness (i.e., their confidence ratings distinguished between correct and incorrect responses). We also examined whether perception of one’s own interoceptive signals or beliefs about such capacity predict the accuracy on the other-HR task. Whilst neither were directly associated to the other-HR task performance, the interoceptive beliefs of those who performed worse at heartbeat counting task determined the performance in the other-HR task (see Fig. 5). Specifically, overestimating one’s own interoceptive capacities (high score on the Interoceptive Accuracy Scale, but low heartbeat counting accuracy) was associated with a worse performance at inferring the heart rate of others. In contrast, underestimating one’s own interoceptive capacities (low Interoceptive Accuracy Scale score, but high heartbeat counting accuracy) did not have such influence. Indeed, the biggest difference in other-HR task accuracy seems to emerge at the high end of the Interoceptive Accuracy Scale (Fig. 4B), where good interoceptive perceivers produce the highest accuracy, while the bad interoceptive perceivers produce the lowest accuracy. This means that deficient beliefs about own interoceptive capacities can have detrimental effects on inferring the cardiac states of other people.

In the original development of the Interoceptive Accuracy Scale (IAS), the authors (Murphy et al., 2020) found that subjective beliefs matched objective interoceptive performance (ability to count heartbeats). In the present study, such correlation was absent, showing that subjective beliefs did not necessarily correspond to objective performance. Another study also failed to find a significant relationship between scores on the IAS and objective ability to perceive cardiac signals (Plans et al., 2021). This lack of association reflects the discrepancy in how well individuals can objectively attune to their heart vs. how well they *believe* they can attune to their heart (Murphy et al., 2019). Indeed, studies find that subjective interoceptive beliefs, rather that objective accuracy, play a role in alexithymia, whereby individuals find it difficult to identify and express feelings (Murphy et al., 2020; Trevisan et al., 2019).

Here, we show that overestimation of interoceptive abilities (i.e., inflated subjective beliefs) can be particularly detrimental.

It is important to note that the current design remained correlational in nature and the statistical significance of the moderating effect of IAcc on the effect of subjective interoceptive beliefs on the other-HR task accuracy remained relatively low (*p* = .04). Therefore, a definitive answer to whether sensitivity to one’s own interoceptive state facilitates sensitivity to the interoceptive state of another person requires further work. For example, a prediction would be that interoceptive training should improve performance on the other-HR matching task. Indeed, while extensive interoceptive training (e.g., via meditation) has been shown to have only a low effect on objective interoceptive accuracy, it was associated with improved interoceptive awareness and/or insight (Khalsa et al., 2008). Because other-HR task performance seemed to rely on interoceptive beliefs, such training could also improve other-HR accuracy.

One could argue that what the other-HR task characterizes is not an ability to infer the underlying cardiac states of others per se but matching the pulsating square to some fluctuations in subtle visual cues that are time-locked to actors’ heartbeats. Indeed, in the debrief majority of participants reported to have relied on facial movements and low-level perceptual changes in the faces of the actors. This may be related to the finding that some actors influenced performance more than others, possibly due to greater facial or low-level perceptual changes (but note that the main inference, the interaction between IAcc and IAS, was present even if these actors were removed; see Section S2). In addition, appearance and perceived health status could also play a role in estimating someone’s heart rate (i.e., more fit people tend to have slower heart rates). Thus, inferring others’ heart rate may also be related to a general perceptual sensitivity or prior beliefs, which were not assessed in the current study.

That said, it is not valid to criticize the other-HR task as being completely unrelated to estimating the heart rate, and only related to estimating some external changes. This is because there is no way to *directly* estimate someone else’s heart rate—interoceptive states are hidden from direct perception. Thus, it is perfectly reasonable that when people try to infer someone else’s heart rate, they base their judgements on minute changes in the person’s appearance or the general state of the person. It may be that the high accuracy on the other-HR task arises due to a better sensitivity to specific cues that reveal another person’s cardiac state. In this case, better interoceptive abilities about one’s own state may sharpen person’s sensitivity to external cues that reflect some interoceptive states but not others. For example, increased heart rate may be related to dilated pupils and increased beathing. What we find, then, is that poor metacognition (i.e., inflated interoceptive beliefs) can have an adverse effect on using any cues to determine the cardiac state of another person.

Nevertheless, future research can further elucidate which exact factors contribute to this ability by considering the possible cues outlined in Fig. 2. Specifically, while the two-alternative forced choice other-HR task reveals whether participants can correctly assign a particular HR trace to its owner, adapted tasks can be used to reveal how and when interoceptive states of others are correctly inferred. For example, a heartbeat tapping task (Ludwick-Rosenthal & Neufeld, 1985; Smith et al., 2021) could be extended to other-HR condition, whereby participants observe our videos and tap along the rate of the observed actor’s heart. Importantly, because the task would allow switching between the self-HR (tap along one’s own heart) and the other-HR (tap along the actor’s heart) conditions while keeping the nature of the task identical, it would allow direct comparison between the sensitivity to one’s own interoceptive signals and the ability to infer the interoceptive signals of others. However, while performance on the heartbeat tapping task has been found to correlate with the performance on the heartbeat counting task, it has been considered a relatively challenging task (Flynn & Clemens, 1988) and the requirement for a motor response (a tap) may disrupt the perception of heartbeats. Another option would be to replace the HR-ownership judgement with a judgement about whether the actor’s HR is decreasing or increasing, which may be a more naturalistic judgement we make in our daily activities (i.e., anger may be accompanied with increased HR). However, the emotionless context used in the present study renders the resting-state HR relatively stable without clear fluctuations that could be estimated by the participants. Thus, combining our paradigm with emotional manipulations would be a fruitful avenue for future studies.

Sensitivity to low-level cardiac cues may be a building block for more complex social representations such as experienced emotions. Indeed, some participants noted their focus on the emotional state of actors when trying to guess their heart rate. Yet, we did not find a statistically significant association between the ability to discriminate the heart rate of others and self-perceived social competence. This conflicts with the view that this ability may be ultimately tied to more successful social functioning, because people who can better infer other’s visceral state would be better at inferring their emotions. However, there is an obvious limitation of using self-report measures to reveal the possession of virtuous qualities. Specifically, socially competent individuals may not necessary perceive themselves to be particularly competent. Moreover, socially incompetent individuals may not know that they are incompetent due to the lack of social competence. Thus, put in real-life situations, people who are better at inferring interoceptive states of others, may be able to utilize these states for social advantage. Studies that tap into *implicit* social competence will be better suited to reveal such a benefit.

This is one of the first studies to quantify interoceptive accuracy in an online experimental context (see also Plans et al., 2021 for a use of PPG-based algorithm in a mobile device). We were able to estimate the heart rates of people via their personal webcam recordings with a photoplethysmography algorithm (rPPG; van der Kooij & Naber, 2019). The heart rate estimates were within 10 bpm consistency across sequential heartbeat counting trials and did not significantly differ across sessions multiple days apart. To quantify the objective interoceptive ability, we used a classic heartbeat counting task (HCT; Schandry, 1981), because, at the time we conducted our study, it was the only interoceptive task that could be implemented online in laptops using just a webcam. Another reason was task length. While the HCT takes approximately 5 min, the phase adjustment task (Plans et al., 2021), for example, would have taken up to 20 min. The more robust multi-interval discrimination task (Brener & Kluvitse, 1988) and the newer heart rate discrimination task (Legrand et al., 2022) would have taken up to 40 min. Because our main task was already 40-min long and because long testing sessions via remote online platforms can result in poor data, adding another lengthy task would have been impractical.

It is important to note that the validity of the HCT has been debated in recent years (e.g., Ainley et al., 2020; Ring & Brener, 2018; Zamariola et al., 2018) and there are several confounds (e.g., knowledge of resting HR) that could have influenced the present results. We controlled for the prior knowledge of the resting HR (Ring & Brener, 1996; Murphy et al., 2018) by including it in our model and showing that interoceptive accuracy was unrelated to the reported resting state HR. Other researchers have also included counting of seconds task as a control, whereby participants are asked to count second instead of their felt heartbeats (Desmedt et al., 2020). The idea is that if participants are attempting to count felt heartbeats, their counting pace should be different to when they are asked to count seconds. We did not include such a control task due to the length of the study. Nonetheless, previous literature has shown that the performance on the HCT is related to neural signatures of interoceptive processing, such as activation within the insular cortex (e.g., Wiebking & Northoff, 2015; Kuehn et al., 2016; Pollatos et al., 2007; Chong et al., 2017) and heart-evoked EEG activity (e.g. ,Pollatos & Schandry, 2004; Coll et al., 2021), suggesting that performing this task engages interoceptive areas of the brain and related electrophysiological signatures. Importantly, right insula damage decreases interoceptive accuracy as measures with the HCT (Ronchi et al., 2015). Lastly, performance in the heartbeat discrimination task has been recently found to represent the strongest, most stable predictor of performance in the HCT (Schulz et al., 2021; but see Hickman et al., 2020). Thus, while no single interoceptive measure is free of confounds, given the online implementation of our study and the aforementioned evidence, we believe that performance in HCT does reflect at least some interoceptive related processes.

Our sample was restricted to younger individuals (up to 40 years old). We did not ask participants to report medical, psychiatric, or neurological disorder history. We did, however, control for self-reported body mass index and resting state heart rate. Thus, while our study suggests that people with poor interoceptive insight tend to be worse at inferring the interoceptive states of others, we cannot draw any conclusions about the relation between interoceptive insights and mental health. Future studies should further investigate the contribution of medical, psychiatric, or neurological conditions that may impact inteorception.

In conclusion, along with Galvez-Pol et al. (2022), the present study has provided further evidence for a capacity to match a heart rate to its owner. This capacity was not directly related to trait-like interoceptive capacity, whereby people who are good at perceiving their own cardiac signals would be good at determining the cardiac signals of others. However, when a conflict arose between the subjective and objective own interoceptive performance, whereby a person believed to possess good interoceptive abilities but did not, the ability to distinguish between others’ cardiac states deteriorated. This fits well with the idea that the metacognitive awareness of our own interoceptive states shapes the perception of such states in other people. Yet, what is the functional role of inferring the interoceptive states of others, and whether it also influences our emotion recognition capabilities or social competences needs to be further investigated.

## Supplementary Information

ESM 1 (DOCX 939 kb)

## Additional Information

### Data and Code Availability

The study design was pre-registered (https://osf.io/jkwp7) and the data along with the analyses codes is publicly available on this OSF project page: https://osf.io/mp3cy/.
