# Supplementary material for: Seeing Through Each Other’s Hearts: Inferring Others’ Heart Rate as a Function of Own Heart Rate Perception and Perceived Social Intelligence
Source: Affect Sci. 2022 Nov 2;3(4):862–77. doi: 10.1007/s42761-022-00151-4 (PMC9743902; doi:10.1007/s42761-022-00151-4)
Supplement: Supplementary file 1 — (DOCX 939 kb) [file 42761_2022_151_MOESM1_ESM.docx]

Seeing through each other’s hearts: Inferring others' heart rate as a function of own heart rate perception and perceived social intelligence

# Supplemental material

Irena Arslanova^1^*, Alejandro Galvez-Pol^3^, James Kilner^4^, Gianluca Finotti^1^, Manos Tsakiris^1,2^

^1^ Department of Psychology, Royal Holloway, University of London, UK

^2^ Centre for the Politics of Feeling, School of Advanced Study, University of London

^3^ Psychology Department, University of Balearic Islands, Spain

^4^ Institute of Neurology, University College London, UK

* corresponding author: [irena.arslanova@rhul.ac.uk](mailto:irena.arslanova@rhul.ac.uk)

## S1: Performance on the IAS (Interoceptive Accuracy Scale) and SI (Social Intelligence scale)


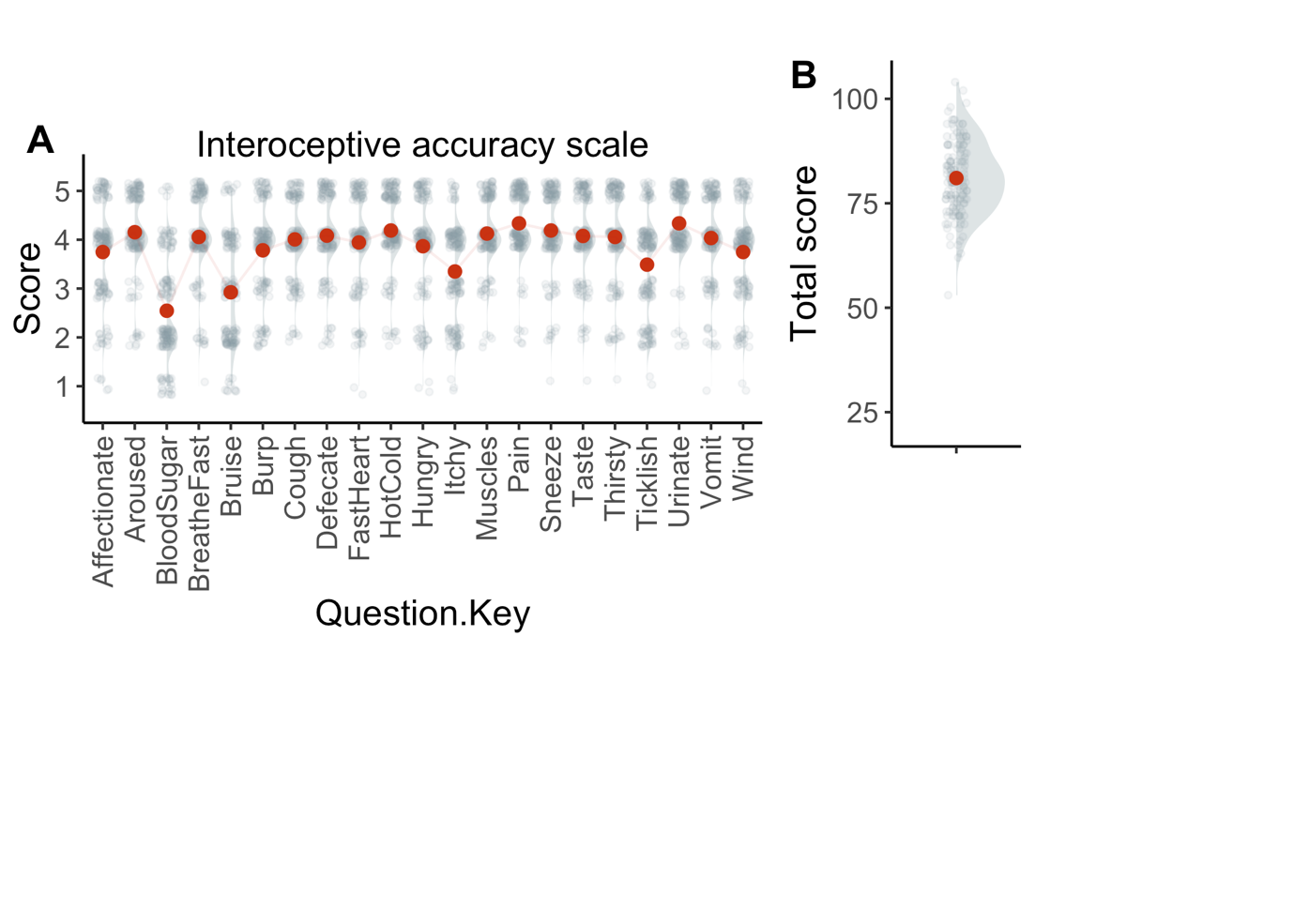


**Figure S1.1.** Group-level (n = 143) scores on interoceptive accuracy scale (Murphy et al., 2020), broken down by each of 21 items (**A**) and aggregated into a total score (**B**).

| **Table S1:** Tromsø’s Social Intelligence Scale (SI) |
| --- |
| 1. I can predict other peoples’ behavior. |
| 1. I know how my actions will make others feel. |
| 1. I understand other people’s feelings. |
| 1. I understand other’s wishes. |
| 1. I can often understand what others are trying to accomplish without the need for them to say anything. |
| 1. I can predict how others will react to my behavior. |
| 1. I can often understand what others really mean through their expression, body language, etc. |
| 1. I have often hurt others without realizing it. |
| 1. I find people are unpredictable. |
| 1. I often feel that it is difficult to understand others’ choices. |
| 1. It seems as though people are often angry or irritated with me when I say what I think. |
| 1. People often surprise me with the things they do. |
| 1. Other people become angry with me without me being able to explain why. |
| 1. I am often surprised by others’ reactions to what I do. |
| 1. I often feel uncertain around new people who I don’t know. |
| 1. I fit in easily in social situations and meeting people for the first time. |
| 1. I am good at entering new situations and meeting people for the first time. |
| 1. I have a hard time getting along with other people. |
| 1. It takes a long time for me to get to know others well. |
| 1. I am good at getting on good terms with new people. |
| 1. I frequently have problems finding good conversation topics. |
| \| Social information processing \| Social awareness \| Social skill \| \| --- \| --- \| --- \| |


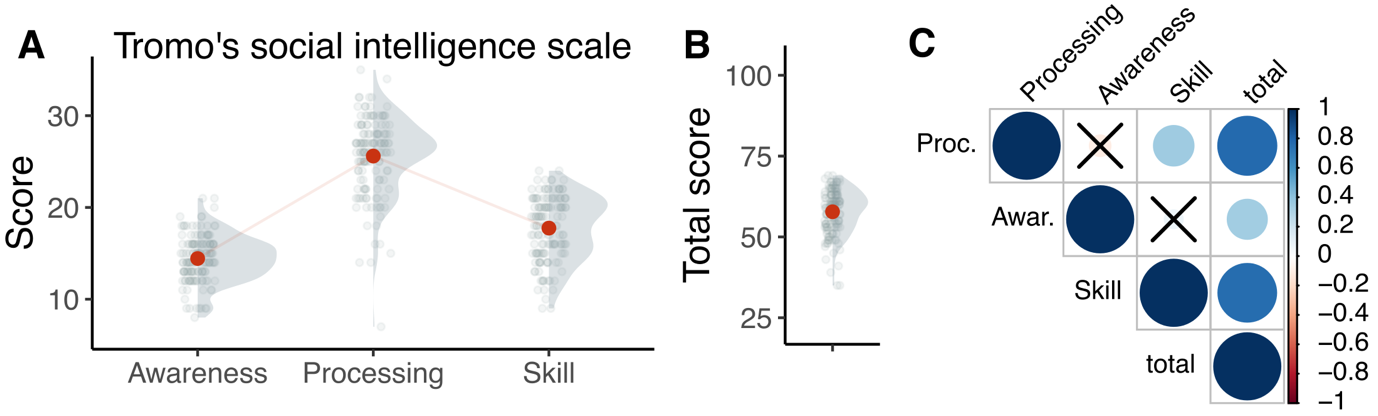


**Figure S1.2.** Group-level (n = 143) scores on Tromsø’s Social intelligence scale (Silvera et al., 2001), **A**) broken down by the 3 subscales (awareness, processing, skill; **B**) and aggregated into total score. **C**) Correlation matrix between the subscales and total score. Crosses indicate non-significant correlation.

## S2: Did specific actors bias task performance?

While the absence of a bias in the main analysis indicated that participants did not show a significant propensity to choose either a right or a left response, it is more interesting to ask whether participants were biased towards a particular actor. For this, we created seven separate datasets, where each of seven actors was paired with another actor (e.g., all combinations where actor A was paired with actors B, C, D, E, G or H). We then re-coded left vs. right response to whether it corresponded to the target actor of that dataset (e.g., actor A) or the other actors (B, C, D, E, G or H). Repeating this for every actor pair (i.e., actor B against all other actors, actor C against all other actors, etc.) provided us with accuracy, d prime, choice bias, and mean reported confidence for the set of trials where specific actor was presented against trials where the specific actor was never presented (**Figure 3**). We then compared these indices with a one-way repeated ANOVA.

In terms of accuracy and d prime, there was a significant effect of actor (F(6,852) = 18.9, p < .001, np2 = 0.1 and F(6,852) = 20.9, p < .001, np2 = 0.1, respectively). A follow-up Bonferroni-corrected tests showed that trials that had actor C consistently produced higher performance than trials that did not have actor C (all p values < .001). Interestingly, analysis on choice bias also showed a significant effect (F(6,852) = 5.5, p < .001, n = 0.04). One sample-tests showed a significant bias away from actor D (t(142) = 4.3, p < .001) and a significant bias towards actor G (t(142) = -3.3, p = .001). This means that in trials were actor D was presented and in trials where actor G was present, participants started to show biased performance. They tended to not select actor D even in pairs when the heart belonged to actor D, whereas they tended to select actor G even when the heart did not belong to the actor G. The actor effect was also significant for mean confidence (F(6,852) = 12.5, p < .001, n = 0.1). Although corrected pairwise tests did not show a consistent pattern, visually, confidence judgements seem higher on trials when actor C was presented. Thus, while actors D and G seemed to bias performance, actor C seemed to improve accuracy accompanied by higher confidence. Further investigations of how these specific actors differ from other actors (e.g., movement, emotional state) are warranted. Note that in the original paper, Galvez-Pol et al., (2020) showed that accuracy was correlated with the difference between the heart rates of the two presented actors. Thus, it may be that the biases observed with specific actors may be due to their specific heartrate (e.g., very slow heartrate that is easily distinguishable or a changing heartrate that is difficult to distinguish).

Importantly, removing those actors from the data still showed a significant contribution to the other-HR accuracy from the interaction between interoceptive accuracy (IAcc) and scores on subjective interoceptive beliefs (IAS; beta = 0.23, p = .04). This suggests that inferring heart rates for actors that were not clearly distinctive from other actors relied on the relationship between observers’ own interoceptive abilities and subjective beliefs about those abilities.


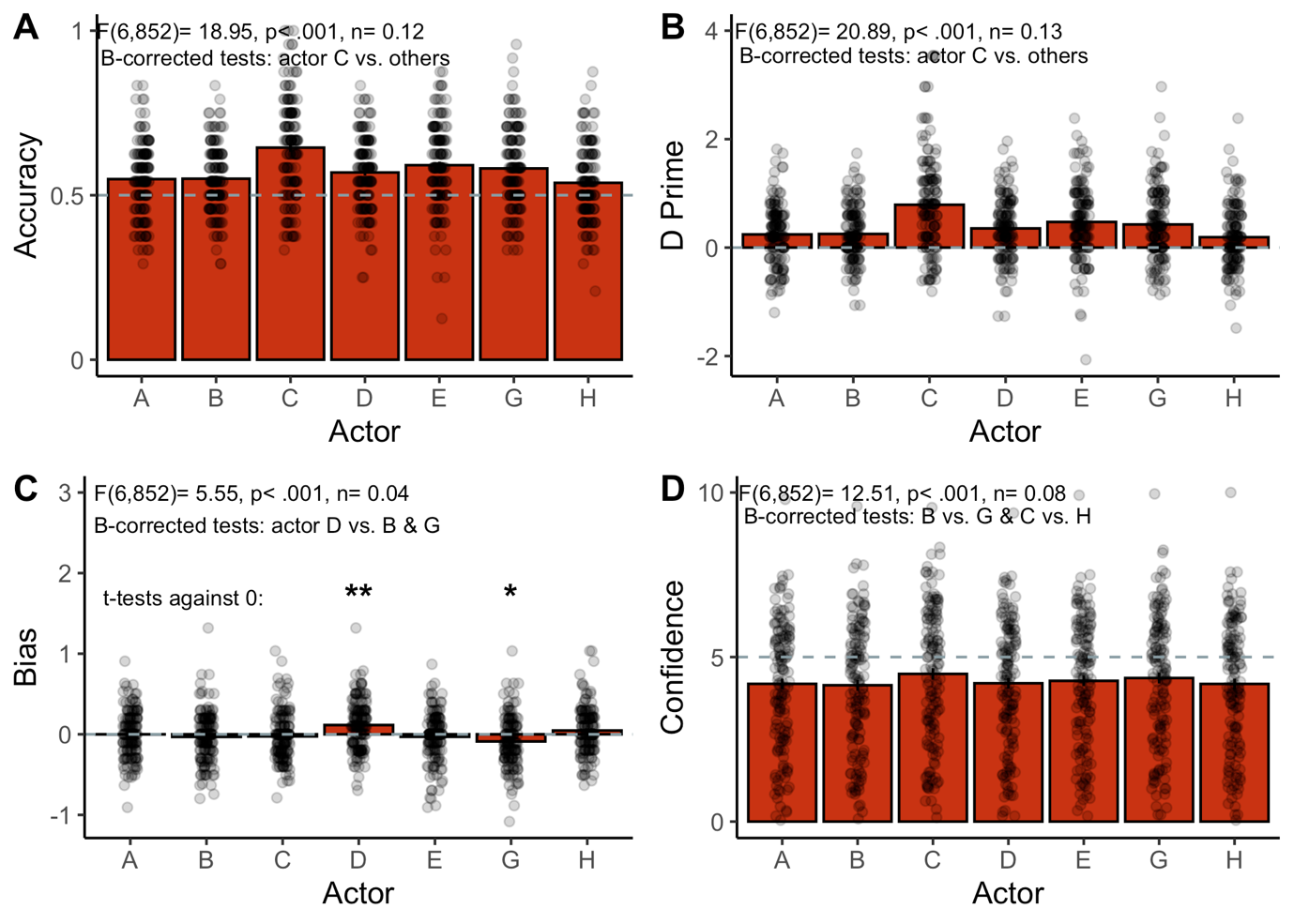


**Figure 3. Performance broken down by presence of a specific actor.** **A**) Accuracy as the proportion of correct responses. **B**) D prime reflects bias-free discriminability (i.e., accuracy independent of propensity to choose a specific actor). **C**) Bias or the propensity of choosing specific actor. Negative values indicate a stronger bias towards the specified actor, whereas positive values indicate stronger bias away from the specified actor. T-test indicates a difference against 0 (absence of a bias, * < .05; ** < .001). **D**) Mean confidence rating. Statistics refer to one-way repeated-measures ANOVA with factor, Actor. Significant main effect was followed by Bonferroni-corrected pairwise tests.

## S3: Which strategy to infer the actors’ HR was most prevalent?

In the main manuscript, we categorized each participants’ response to the question, “*Could you tell us which cues from the videos did you use to perform this task*?” to one of 8 categories (general movement, breathing, person’s state, eye movements, association, skin colour, pulse at neck, physique), including “none” when they did not provide any prompts, or their prompts were unclear, but some of them referred to general intuition. This gave us some idea on whether relying more heavily on one strategy results in better performance as compared to relying mainly on another strategy. However, it did not show which strategy was more prevalent as many participants implied the use of multiple strategies. Thus, we reanalysed the prompt use by taking each of the 8 categories, plus an additional category coding for “intuition” and calculating the sum of times it was mentioned (even by the same participant). For example, if a participant reported: “*I looked at eye movement and body language, also i tried to look at their breathing*”, it would result in 1 mention of “eye movement”, 1 mention of “general movement”, and 1 mention of “breathing” (please refer to the main manuscript for descriptions of what each category represented). **Figure S3.1** shows how many times each category/strategy was mentioned. Most prevalent mentioned strategies were looking at either eye movements (e.g., blinks, movements), general movements (e.g., head movements, twitching, swallowing), or breathing (e.g., breathing movements); next person’s state (e.g., whether they were nervous or calm, their facial expression); then skin colour or pulse at the neck; then people mentioned trying to form associations between specific heart rates and actors; finally some mentioned physique and few mentioned relying on general intuition.


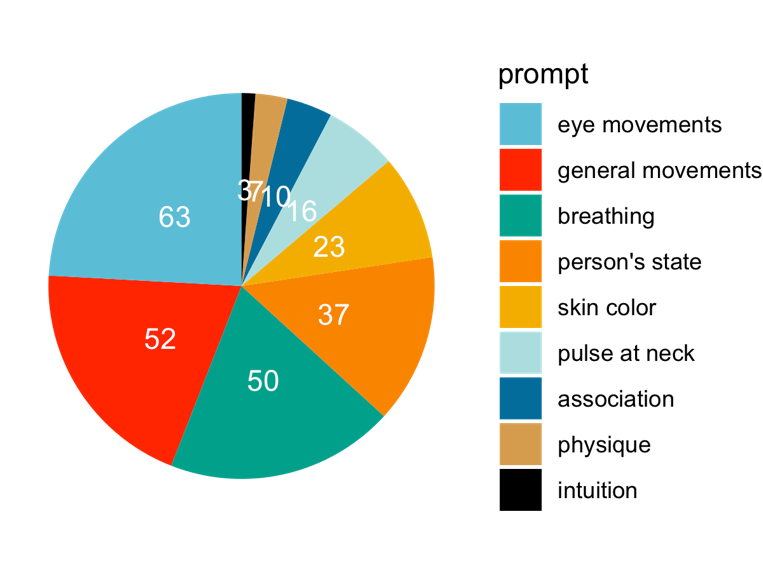


**Figure S3.1.** How many times each prompt was used to describe which cues participants used to perform the task.
